# Acute phosphine poisoning on board a bulk carrier: analysis of factors leading to a fatal case

**DOI:** 10.1186/s12995-015-0050-0

**Published:** 2015-03-01

**Authors:** Brice Loddé, David Lucas, Jean-Marie Letort, Dominique Jegaden, Richard Pougnet, Jean-Dominique Dewitte

**Affiliations:** European University of Brest, EA 4686 – CS 93837 – 29238, Brest, Cedex 3 France; Occupational and Environmental Diseases Department (Service de Santé au Travail et Maladies liées à l’environnement) CHRU Morvan, 2 avenue FOCH, 29609 Brest, Cedex France; Emergency Department and Maritime Emergencies (Service d’urgences et SAMU de coordination médicale maritime), CHRU La Cavale Blanche, Boulevard Tanguy Prigent, 29200 Brest, France; Occupational Health Service in Iroise (Service de santé au travail en Iroise), 37, rue Voltaire, 29200 Brest, France; French Society of Maritime Medicine (Société Française de Médecine Maritime), 22, Avenue Camille Desmoulins, 29200 Brest, France; European Society for Environmental and Occupational Medicine, Hamburg, Deutschland

**Keywords:** Phosphine, Poisoning, Seafarer, Death, Bulk carrier

## Abstract

**Objectives:**

To determine accidental factors, clinical presentation and medical care in cases of seafarers presenting phosphine poisoning symptoms on board a bulk carrier.

To consider primary prevention of this pathology, which can have extremely severe consequences.

**Methods:**

To analyse circumstances resulting in toxic exposure to phosphine in the sea transport sector.

To obtain information from medical reports regarding the seafarer’s rescue.

To identify the causes of this accidental poisoning and how to establish an early, appropriate diagnosis thus avoiding other cases.

**Results:**

In February 2008, on board a bulk carrier with a cargo of peas, a 56-year-old seafarer with intense abdominal and chest pains, associated with dizziness, was rescued by helicopter 80 miles away from the coast. Despite being admitted rapidly to hospital, his heart rate decreased associated with respiratory distress. He lost consciousness and convulsed. He finally died of pulmonary oedema, major metabolic acidosis and acute multi organ failure.

The following day, the captain issued a rescue call from the same vessel for a 41-year-old man also with abdominal pain, vomiting and dizziness. The ECG only revealed type 1 Brugada syndrome.

Then 11 other seafarers were evacuated for observation. 3 showed clinical abnormalities.

Collective poisoning was suspected.

Medical team found out that aluminium phosphide pellets had been put in the ship’s hold for pest control before the vessel’s departure. Seafarers were poisoned by phosphine gas spreading through cabins above the hold. It was found that the compartments and ducts were not airtight.

**Conclusion:**

Unfortunately, a seafarer on board a bulk carrier died in 2008 because of acute phosphine poisoning. Fumigation performed using this gas needs to be done with extreme care. Systematic checks need to be carried out before sailing to ensure that the vessel’s compartments are airtight.

## Introduction

The chances of surviving after being poisoned on board are much less favourable than on shore [1]. This difference in prognosis can be explained by several factors. Other than the obvious transport difficulties and medical assistance which can be a long way from the coast, it is often the vessel’s architecture in a hostile environment which makes both preventive and therapeutic treatment difficult.

We are going to describe the problem areas responsible for the cases observed on board a bulk carrier navigating off the coast of Finistère in France, in February 2008. On this date, the captain called emergency rescue services for one of his crew members who was suffering from abdominal pain and difficulties breathing. He was evacuated by helicopter but died shortly after. Several hours later, another emergency call was made for a similar clinical case to the previous one. This seafarer was also evacuated by helicopter. The vessel was rerouted to the nearest port facilities and the eleven other seafarers were taken to hospital for observation. Facing multiple cases like this, collective poisoning was investigated and the chemical agent suspected and later identified was phosphine.

In order to determine accidental factors and to consider primary prevention of this poisoning which occurred on board, we have analysed circumstances resulting in toxic exposure to phosphine in the sea transport sector.

Discussions about this case have lead to comparisons being made with other cases in different circumstances. Nevertheless, we focused on unintentional poisoning [2,3] even if voluntary ingestion is frequently reported [4,5].

## Case report

### History and medical care

**Figure 1:**
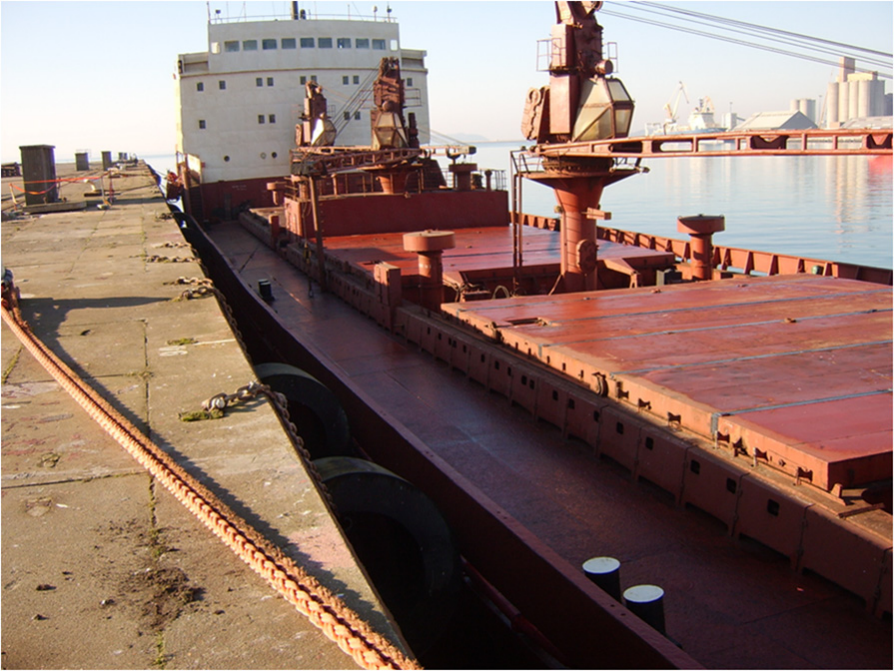
**Bulk carrier where the poisoning occurred.**

On 7th February, 2008 at 18:11, a Romanian bulk carrier (Figure 1) navigating 80 km north of the island of Ouessant, sailing from Rouen in France to Cairo in Egypt, with a load of approximately eighty-thousand tonnes of peas, contacted the Maritime Rescue Coordination Centre (MRCC) Corsen to evacuate of one of its crew members for medical reasons (*patient n° 1).* This 56-year old man, discretely over weighted, onboard electrician, had received orders from the captain to rest in his cabin since the previous evening as he had been suffering from increasingly severe digestive disorders in the form of acute abdominal pain accompanied with dizziness. His was given emergency medical care and was taken by helicopter to Brest Hospital, France. No information was given by the officers on the vessel as to possible poisoning. No other seafarer was reported ill in the first report.

The first medical examination found the patient conscious and oriented. The Glasgow Coma Scale (GCS) score was 15 at this time. He complained of abdominal and chest pain similar to symptoms of an acute coronary syndrome associated with high blood pressure and reduction of the diastolic-systolic time interval. It was not clear from the ECG done in the helicopter whether there was myocardial infarction or not. In the Accident and Emergency waiting room, the patient’s respiratory condition with cyanosis deteriorated. Suddenly, he convulsed, showed signs of unconsciousness which deteriorated rapidly justifying sedation and intubation. An emergency chest x-ray showed an oedema spreading throughout the two lungs and the heart. An electro-mechanical dissociation was found. Closer inspection of the ECG readings excluded myocardial infarction. The patient went rapidly into shock: his pulse and blood pressure were impossible to take, despite volume expansion and administration of adrenaline, dobutamine and atropine. Biological results were the following: CBC normal, PT at 61% with no other coagulation abnormality, lactic acidosis (anionic hole 39, lactates 25.5 mmol/L) decompensated by an alkaline reserve down to 7 mmol/L), and hyperglycaemia 4.6 g/L, CPK and troponine were both normal. No abnormalities were found in the hepatic or pancreatic results either.

The seafarer died at 21:35 as a result of these major acid-base abnormalities. Metabolic acidosis was suspected to have been caused by an external factor with no further details. The MRCC was informed of the death.

The following morning at 7 :23, the cargo ship (having continued its route to Egypt), and at that time 60 kms from the coast, made another call to the authorities to evacuate a second seafarer *(patient n° 2),* 41 years old, who was also had symptoms of dizziness with repeated vomiting and abdominal pain. The MRCC requested medical evacuation by helicopter. Collective poisoning was by then suspected.

The medical team found the man to be conscious and oriented. GCS score was 15 at this time, 96% saturation in ambient air. He was complaining of abdominal pain, vomiting and dizziness when standing up and his condition had been deteriorating since the previous evening. Symptoms improved considerably when the seafarer left his bunk. His medical history was the following: active smoker, appendectomy, frequent abdominal pain.

At the same time, the Marine Prefecture services requested the cargo ship to head for the coast for inspection. A further conversation with the ship’s captain concerning the chemical products on board lead the authorities to suspect collective phosphine poisoning: phosphine had been used as an insecticide on board the vessel in the holds containing peas. Clinical examination of the second patient was normal and the biological results were completely normal. Moreover, there was no acid-basic imbalance or any other gasometric abnormality, or higher cardiac enzyme or hepatic readings. The ECG showed a rise in the ST segment in V_1_ V_2_ associated with a right bundle branch block, characteristic of a type 1 Brugada syndrome. This patient was non-Asian and had no history of sudden death in the family. The seafarer, at that time, with no symptoms, was admitted to hospital, into cardiac intensive care for monitoring for a documented risk of total atrioventricular block or heart rhythm disorders such as ventricular tachycardia. Three days later, he left hospital with stable ECG readings. Onboard the ship, while waiting for the chemical diagnosis, the seafarers were kept out of danger on deck.

As there was no risk of explosion as initially suggested, the ship was authorized to go into the bay of Brest and berth in the port. The eleven other crew members were taken to hospital for medical examination.

Amongst these eleven seafarers aged between 34 and 59 years old, nine said nothing was wrong, one complained of weakness and the other complained of chest pain which improved when taken away from the contaminated area. As well as a standard clinical examination, each patient had a biological examination, chest x-ray and ECG. Specific clinical information being looked for in these consultations and examinations were the following: any medical history, presence or not of neurological signs (headaches, dizziness and abnormalities in the neurological examination); digestive signs (pain, nausea, diarrhoea…); difficulties breathing and chest pain. The biological examination included renal, hepatic, troponine, lactatemia and plasma ionogram testing.

The patient complaining of weakness (*patient n°3*, 46 years old) was the one who confirmed the death of his colleague. At that time, he was wearing a protection mask. Clinical examination in Accident and Emergency was completely normal. Oxygen saturation was 95% in ambient air with spontaneous breathing even though a smoker. Para-clinical examination was also normal. He was kept under observation for twenty-four hours.

The patient complaining of occasional chest pain on board the vessel (*patient n° 4*, 38 years old) improved considerably in Accident and Emergency. Symptoms including retrosternal chest pain had lasted for several minutes the previous day. Clinical examination was normal and the ECG showed a banal right bundle branch block. He was kept in for observation for twenty-four hours.

Another patient (*patient n°5*, 55 years old and ship’s cook), had no symptoms, but showed some abnormalities when examined. Chest examination found crackling at the base of the lungs and the ECG showed up a right bundle branch block. His biological results were completely normal and the chest x-ray didn’t show any signs of overload. He was kept under observation for twenty-four hours. At the end of the observation period, the branch block had disappeared and the chest examination had become normal. He was advised to consult again quickly should palpitations, difficulties breathing or chest pain reoccur in the following few days.

For the eight other patients, clinical examinations, biological results, chest x-rays and ECGs were normal. They were, however, kept for observation for twenty-four or forty-eight hours (according to a calculated exposure risk), except one of them who refused to go to hospital and for another whose presence on board was deemed necessary and compatible with his state of health.

### Medical follow-up

Out of the 11 seafarers on board, 10 are still alive and in good health 5 years after this tragedy.

An autopsy was carried out on the deceased seafarer on 13 February 2008, 6 days after his death. A dilated heart condition was detected, a healthy coronary network, absence of lung tissue abnormalities and pleuropericardial effusion. The liver, spleen, kidneys (other than an isolated cyst), encephalon and alimentary canal were normal. Moreover, there was no evidence of trauma. Blood, stomach, kidney, brain, heart and lung samples were taken for anatomical, pathological and toxicological analysis.

From a histological point of view, the tissues analysed were within the normal range. No traces of medication or narcotics were found. Phosphorous compounds were tested using a technique of gas phase analysis associated with mass spectrometry (detection from 0.1 mg/l). The Musshoff [6] technique was used for phosphine calibration. In all tissues, phosphine characterisation was negative. It was extremely likely that the phosphine had been eliminated from the samples considering its high volatility [7] or due to spontaneous ignition [8], bearing in mind that it was tested for 6 days after death and that it was absorbed by inhalation and not ingestion. To conclude, when exposure and clinical symptoms are well identified, chemical analysis for phosphine in blood or urine is not recommended as phosphine is rapidly oxidised into phosphite and hypophosphite [8,9].

### Toxicology tests

Phosphine is a gas used for the fumigation of grain on board cargo ships [1]. In this specific case, an external French company was responsible for fumigating the vessel. This company started procedures by offloading the product on the quayside in the port of Rouen, before the vessel’s departure.

Once the grain had been loaded into the cargo holds, it was then treated with aluminium phosphide pellets placed on the surface of the piles of grain in each hold: equivalent of 1 g to 1.5 g of aluminium phosphide per m^3^. This large quantity of aluminium phosphide pellets reacted in contact with the air, water and moisture to release a phosphine gas thereby killing insects and larva.

The chemical reaction is AlP + 3 H20 → PH3 + Al(OH)3 [6].

In other words, the more humid the weather (which is the case in February in Europe) the faster (and at high levels of concentration) gas is produced.

**Figure 2:**
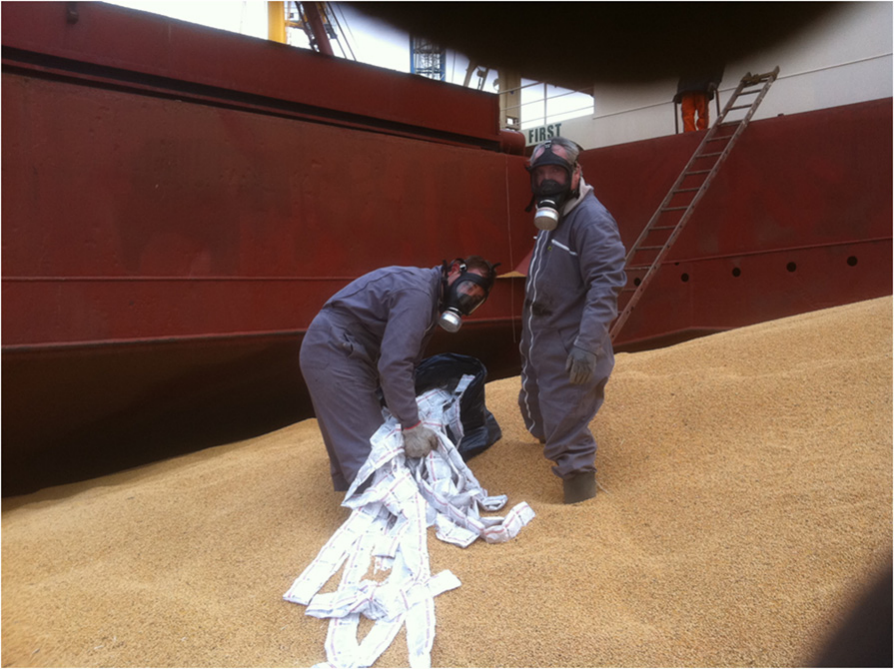
**Operators handling blisters which contained aluminium phosphide pills.**

Phosphine is an expanding gas which spreads through the closed environment of holds reaching pesticide concentration (several hundred ppm). This concentration reaches its peak once all the aluminium phosphide pellets have reacted with the air or water in the holds then it decreases during the voyage, until hardly detectable after 3 weeks. Nevertheless, great care must be taken when handling the open pellet blisters after fumigation in order to control complete dissolution. Protective equipment must be worn in these cases (Figure 2).

The ship’s captain was informed of this fumigation and the external French company gave him MSA colorimetric tubes so that he could test for the presence of this gas onboard should the gas spread from the hold through the ship.

In the case we are presenting, phosphine gas was tested for on 8 February, in other words, the day the second seafarer was poisoned. Testing was positive in the two cabins of the poisoned seafarers. Emergency services were aware of these positive tests and told the ship’s captain to evacuate and ventilate the two cabins.

Marine rescue services arrived onboard 4 hours after the cabins had been ventilated and found 0.24 ppm of phosphine near the holds and a trace of phosphine in the ventilated cabins.

## Analysis and discussion

The first written review on acute phosphine poisoning dates from 1958 [10] and the first recorded case dates from 1900. Numerous deaths have been reported: when using the chemical product; during the chemical manufacturing process [11]; when it started to be used (including the maritime sector) [12,13] and in cases of voluntary poisoning [14-17]. However, to our knowledge, these are the only cases on board a bulk carrier. There are, however, numerous cases on board container vessels [12,13].

In fact, aluminium phosphide is used as a rodenticide, insecticide and fumigant for stored cereals. Agriculture and transport are therefore professional sectors where there is potential exposure [18]. Peshin and Coll also showed a high incidence of poisoning due to household pesticides compared with agricultural pesticides, clearly emphasizing the need for educating and generating awareness about correct usage as well as implementing prevention programmes [19]. Unfortunately, this ready access also means that aluminium phosphide has been listed as a commonly ingested toxin for attempting suicide [20].

Random exposure is uncommon. It can be due to inhaling the still-expanding gas in food storage [21] or when ingesting food already contaminated by this chemical product [22,23]. Veterinarians caring for animals [24] and health workers caring for people who have absorbed aluminium phosphide can also come into contact with generated phosphine gas [25].

Until now, case reports as well as anatomical, pathological post-mortem studies have not completely identified the pathophysiological phenomena involved [26-29], even if animal experimentation seems to point to mitochondrial respiratory chain inhibition due to cytochrome C oxydase damage in humans. Moreover, inhibition of other detoxification enzyme systems may also explain why system damage is so severe [30,31]. It is also likely that the unclear clinical picture, especially in cases of low exposure, has contributed to not being able to identify the true cause of this problem in numerous cases.

In the above case, several factors were responsible for the seafarers severe poisoning. Firstly, when the vessel was at berth, aluminium phosphide pellets were put down by subcontractors and not the ship’s crew. The seafarers were unaware of insect fumigation: no details or explanation had been given to them about it. Moreover, only the captain knew what kind of product had been used while the rest of the crew was unaware of how poisonous it was.

Information and training on phosphine gas were, therefore, insufficient.

Secondly, phosphine gas expansion rapidly reached pesticide concentration in the holds due to high moisture content in this large, closed area at this time of year (February is a winter month in Western Europe). As a result, for 1.500 g used per m3 (common practice), the concentration of phosphine gas reached 700 ppm inside the holds as gas concentrations expanded and levelled off throughout the closed compartments. It was estimated that at peak concentration in the holds, approximately the same concentration of gas would have been found in the contaminated cabins.

Thirdly, gas had leaked had from the holds to the cabins. Several months after this incident, the expertise highlighted the causes of this leak. Moreover, Lloyds© found two main problems.

The first problem revealed holds which were no longer airtight due to rusting of certain partitions. The second related to the smoke detection system between the holds and the crew quarters (in particular the deceased seafarer’s cabin). Initially shut off, the ducts were reopened because the crew wasn’t aware of potential fumigation in the holds. This then meant that the gas could expand throughout these closed areas (firstly the holds, then the cabins). Therefore, the spreading of phosphine from the holds towards the quarters, located on the deck above could be explained by the fact that the ventilation ducts connecting the two areas of the vessel were not shut off.

In order to explain why Patient 1 was the first and the most severely intoxicated, we found that exposure was higher in his cabin and he stayed in there longer. In fact, his cabin was found to be the one where ducts were completely reopened. Moreover, the captain told him to stay in his cabin when he began to present the first symptoms. Meanwhile, the other crew members were at work in different parts of the boat (so not in their cabins). When Patient 1 closed the door for a rest it caused the gas to expand in this completely airtight area.

Fourthly, the non-specific symptoms of the first affected seafarer looked like an abdominal surgical emergency. This delayed the diagnosis of acute poisoning and was only confirmed after the second case. In addition to all this were problems of communication between the captain and coastal rescue services.

Fifthly, as the ship was far out at sea, considerable time elapsed between rescue services receiving the calls and arriving on the scene. And, unfortunately, despite rescue services removing the first poisoned seafarer from the area and giving him medical care, the lesions were far too advanced to be able to save his life.

Finally, phosphine was only detected after the event, with information given by the captain about pellets used to fumigate the holds. Moreover, there is still no antidote for phosphine despite the fact that protective treatment is being discussed [26].

This additional case of poisoning raises questions about the extensive use of phosphine in transport of grain by sea, combined with often badly maintained fleets. If, in most cases, there is no choice other than using smoke insecticides, it must be used by people who are trained and qualified in risk management.

Unfortunately, the Californian study by Melher and Coll [31] confirms that in over 75% of cases of poisoning, those affected are the ones who are not trained in fumigation processes. In this particular case, seafarers responsible for the ship’s operations had little or no training in risks related to phosphine.

Maintenance of ships and, in particular, the holds, is of the upmost importance; as well as training in risks related to working at sea, including the use of colorimetric detection kits.

From a medical angle, this case also stands out:

- Patient n° 1 showed digestive symptoms associated with dizziness followed by chest pain after being exposed for several hours. These symptoms are classic. His condition then deteriorated abruptly and he died of multi-system organ failure with major metabolic acidosis. It was difficult to determine the exact causes of death: was it linked to acute heart-respiratory failure or rather the result of the acid-base imbalance? Mechanisms of metabolic acidosis in phosphine poisoning are thought to be the cause of oxidative stress [26], and considering the severity, could well be the direct cause of death in this case. This has already been described, but does not characterise the most common cause of death which is ARDS. However, we can assume that metabolic acidosis was secondary to pulmonary-heart failure, especially since the acids responsible were of endogenous origin (lactates). We also noted heart damage with myocardial dysfunction with electro mechanic dissociation (direct result of poisoning or a case of multi organ failure).
- Patient n° 2 showed cardiac transmission disorders. These are also frequently described, but in atypical cases with ST segment elevation characteristic of a type 1 Brugada syndrome, which improved after a few days. Functional symptoms were similar to patient n° 1. This symptoms could be compared with those described in case of weak occupational exposure in Misra and coll study [32].
- As for the other seafarers, it is hard to estimate or even to appreciate the reality of this kind of poisoning. The only abnormality found was a right bundle branch block which spontaneously improved after several hours of monitoring, with no functional troubles.

## Conclusion

If the transport of grain by bulk carrier cannot be done without the use of insecticides, strict security measures must be enforced. The advantage of phosphine is that it is relatively simple for trained personnel to use. However, incidents or accidents due to exposure are increasingly common because global tonnage of grain transported by sea is rising every year. Safety procedures must be scrupulously respected to ensure the safety of the seafarers on board, and this will require appropriate training of personnel. Particular attention must be paid to: on board ventilation; storage of aluminium phosphide pellets; airtight holds and access to a simple, reliable way of detecting this gas on board.

From a medical point of view, the doctor examining the seafarer with non-specific symptoms as described above should suspect phosphine poisoning; then, taking into account any medical history, should actively look for the possible source of poisoning. On one hand, the risk to the other crew members should be checked and they should be kept way from danger. On the other hand - even if there is no effective antidote - clinical improvement of the poisoned patient can be expected and paraclinical examinations within this context can be carried out.

As there is no specific treatment and taking into consideration current knowledge on this subject (even if types of protective treatment are under discussion), only symptomatic treatment to sustain vital organ function is possible. When stabilised, the patient should be observed and the heart monitored. This observation period should last at least 72 hours. Afterwards, biological monitoring for renal and hepatic function should be done, especially if the initial results are abnormal.

## Consent

Written informed consent was obtained from the patients for the publication of this report and any accompanying images.
